# Effects of Time of Initial Exposure to MSV Sarcoma on Bone Induction by Dentine Matrix Implants and on Orthotopic Femora

**DOI:** 10.3390/ijms11093277

**Published:** 2010-09-15

**Authors:** Krzysztof Włodarski, Paweł Włodarski, Ryszard Galus, Aniela Brodzikowska

**Affiliations:** 1 Chair of Histology and Embryology, Institute of Biostructural Research, Medical University of Warsaw, Chalubinskiego 5 Str., 02-004 Warszawa, Poland; E-Mails: pwlodar@wum.edu.pl (P.W); rkgalus@wp.pl (R.G); 2 Department of Conservative Dentistry, Institute of Stomatology, Medical University of Warsaw, Miodowa 18 Str., 00-246 Warszawa, Poland; E-Mail: aniela.brodzikowska@wum.edu.pl (A.B)

**Keywords:** ectopic osteogenesis, murine demineralized incisors, MSV sarcoma, periosteum activation

## Abstract

HCl-demineralized murine lower incisors were implanted intramuscularly into syngeneic BALB/c mice to induce heterotopic osteogenesis. Implants were exposed at the early, preosteogenic stage (4), or at the later, osteogenic stage (12) to the Moloney sarcoma virus (MSV), which within 3–4 days results in a sarcoma. The yield of bone induction was determined by weight of dry bone mass following NaOH hydrolysis of soft tissues. To verify the effect of this sarcoma on orthotopic local femoral bone, the dry mass of the tumor-exposed femora was measured and compared with the weight of MSV-unexposed contralateral controls. MSV-sarcoma or cells involved with their spontaneous rejection have a stimulatory effect on the periosteal membrane of the tumor-adjacent femoral bones, increasing their dry mass on average by 18%. No stimulatory effect on heterotopic bone induction was observed when the MSV sarcoma grew during the early, preosteogenic stage (4 onward), but when the tooth matrix had been exposed to such tumor at the already bone-forming stage, (12 onward), the yield of bone induction was enhanced. Thus, it is postulated that lesions induced by MSV during the early, preosteogenic stage inhibit recruitment of osteoprogenitor cells or degrade Bone Morphogenetic Proteins (BMPs) released by matrix resorbing inflammatory cells, whereas when acting on already existing bone they have a stimulatory effect.

## 1. Introduction

In rodents, the development of tumors by intramuscular injection of Moloney sarcoma virus (MSV) triggers a proliferative response of the periosteal membranes in tumor-adjacent bones. In such activated periosteum, proliferation and differentiation of chondro- and osteoprogenitor cells results in cartilage and bone matrix deposition. Thus, the process of periosteal bone formation recapitulates both endochondral and intramembranous osteogenesis, the last type dominating. The newly formed bone collar is firmly attached to maternal bone and is very slowly resorbed by osteoclasts, so in some cases an increment of bone mass, sometimes exceeding 100% of the original mass, persists for several months post sarcoma development [1].

In mice, MSV-induced tumors appear 3–4 days post infection, and grow progressively for 5–10 days, then gradually regress and disappear in most cases by day 16–20. The regression of these tumors are immunological events, mediated mainly by a cellular response [2–6], although a humoral response also contributes to some extent in tumor rejection [4,7].

Demineralized dentine is as efficient an inducer of cartilage and bone progenitor cell proliferation and differentiation as is demineralized bone [8–11]. On implanting into a muscular pouch, demineralized dentine triggers differentiation of host mesenchymal cells to a chondro- and osteogenic lineage. In the model of heterotopic bone formation by tooth matrix, endochondral and mesenchymal-type osteogenesis are present.

Soluble signals released from demineralized tooth matrix belong to the family of Bone Morphogenetic Proteins (BMPs) that belong to the TGF-beta super family of proteins [12–14].

Mechanisms of regulation and maintenance of heterotopically formed bone are not fully understood [15], and there are a number of differences between ectopic and orthotopic bones. For example, ectopic bones lack true, functioning periosteal and endosteal membranes [16]. In human heterotopic ossification, the derived cells exhibited elevated levels of collagen synthesis and alkaline phosphatase activities ascribed to bone forming cells [17]. In general, heterotopic bone is metabolically more active than skeletal (orthotopic) bone. Toom *et al.* [18] found that the expression of BMP-2 and TGF are higher in ectopic bones and Chauvean *et al.* [19] reported strong over expression of osteocalcin mRNA, an event associated with upregulation of osteonectin and collagen type I synthesis.

Demineralized bone matrix also contains proteins which inhibit the BMP activity both *in vivo* and *in vitro* [20]. Also, bone morphogenetic proteins BMP-3 and BMP-7 are regulators of bone inductive activity of demineralized matrix by inhibition of osteoprogenitor proliferation and stimulation of differentiation and expression of bone markers [21,22].

The immune rejection of MSV-induced tumors releases multiple factors influencing bone homeostasis [23]. Isolated human osteoblast proliferation may be either activated or inhibited by inflammatory cytokines, depending on their concentration and duration of exposure [24], whereas adjuvant-evoked granuloma cells activate endosteal osteogenesis [25]. Lesions induced by T-cell mitogen Concanavalin A have increased the yield of demineralized tooth-induced bone [26].

The aim of this study is to examine the timing of MSV sarcoma induction on locally induced bone by demineralized murine lower incisors. In this study, MSV-sarcomas were induced during the early, preosteogenic stage of bone induction (virus injection 4 days post dentine implantation) or at a late stage, when chondro- and osteogenesis is advanced (virus injection 12 days post dentine implantation). The effect of MSV sarcoma on adjacent femoral bone was also monitored in order to compare the effectiveness of lesions on orthotopic and ectopic bones.

## 2. Materials and Methods

### 2.1. Demineralized Incisor Preparation and Characteristics

Four to five-month-old BALB/c inbred female mice were used in our experiment in accordance with the Medical University of Warsaw’s guidelines for the care and use laboratory animals. The mandibles of the killed mice were removed and put into 0.6 N HCl, chilled on ice. After 60 min, the incisors were removed from the alveolar bone and transferred to a fresh HCl solution where demineralization continued for another 3–4 hr, thus the duration of demineralization was 4–5 hr. The necrotic dental pulp in the crown and root of each incisor was removed by squeezing the demineralized tooth. Then incisors were vigorously washed in distilled water and lyophilized.

Demineralization of adult murine incisors for 4 hr in the 0.6 N HCl removes completely their mineral phase. The mean wet weight of isolated incisors demineralized for 4 hr was 4.86 ± 1.0 mg (n = 13) and was equal to the specimens demineralized for 22 hr (mean weight 4.81 ± 0.7 mg, n = 9). Perhaps some traces of mineral remained following the procedure, but did not affect bone induction. The prolonged hydrolysis of murine incisors for 22 hr slightly reduces the yield of bone induction [26].

The 4 hr lyophilized demineralized female mature BALB/c lower incisors are very homogenous. Their average weight was 0.95 ± 0.05 mg (n = 17), thus we considered them as a standard dose of Bone Inducing Principle.

### 2.2. Implantation of Demineralized Incisors

The animals were anesthetized with an intraperitoneal injection containing Rompun (Bayer AG, Leverkusen, Holland) and Calypsol (G. Richter, Budapest, Hungary). After shaving, short (4–5 mm) longitudinal skin and muscle incisions were made on the inner side of the left and right thighs. Lyophilized incisors were inserted into the muscle pockets, the skin was sutured with a 3-0 Dexon “S” polyglycolic acid suture, and the wounds were disinfected with 70% alcohol.

Individual recipient mice received bilateral implants from one set of prepared incisors. Four or twelve days after implantation, the left or right limb was injected with 0.2 mL of standard MSV suspension, the contralateral right or left limb was injected with saline only. On average, half of the animals were injected in left limb, and half were injected in right limb, in order to exclude the influence of manual inconsistencies in the surgical procedure.

The MSV preparation was a saline homogenate of the tumor, grown in a newborn BALB/c mouse following inoculation of MSV. The tumor was harvested two weeks post inoculation. The virus suspension used in these experiments was prepared according to Chanaille *et al.* [27].

Animals were observed daily, and the time of tumor appearance, growth and disappearance was recorded. Animals which did not develop a tumor (5% of cases) were excluded.

Animals were killed by cervical dislocation following CO_2_ inhalation, four weeks following incisor implantation, to assess bone induction yield.

### 2.3. Assessment of Excised Implants and of Femoral Bone

The implants, together with the surrounding thigh muscles, were excised and hydrolyzed overnight in 0.1 N NaOH at 64 °C.

The demineralized incisors and soft tissues were completely dissolved during hydrolysis, without affecting mineralized tissue mass. Thus, the undigested material recovered was solely mineralized component of bone tissue. These undigested deposits were washed in distilled water and dried overnight at 64 °C. The dried mineral was weighed to an accuracy of ±0.1 mg.

The femoral bones from both limbs were excised and hydrolyzed as above. The femoral bone dry mass was measured to the same accuracy, providing information on the effect of MSV sarcoma on adjacent femoral bone. The femoral dry bone mass gain following MSV sarcoma development was expressed as a percentage of dry mass of the contralateral bone not exposed to the tumor.

### 2.4. Histology

The implant and surrounding tissues were fixed in Bouin fixative, decalcified in a saturated solution of EDTA, embedded in paraffin and sectioned at 8 μm. Sections were stained with PAS-hematoxylin and examined with the light microscope.

The number of animals, their division into groups according to the MSV-injection schedule, and the bone induction yields are shown in Table 1.

### 2.5. Statistical Analysis

Student’s t-test was used to test for significant differences between the induced bone yields. Differences were considered significant when the *p* value was less than 0.05. Statistical analysis was performed using SAS V.6.12 for Windows software, (SAS Institute, USA).

## 3. Results

**Figure 1:**
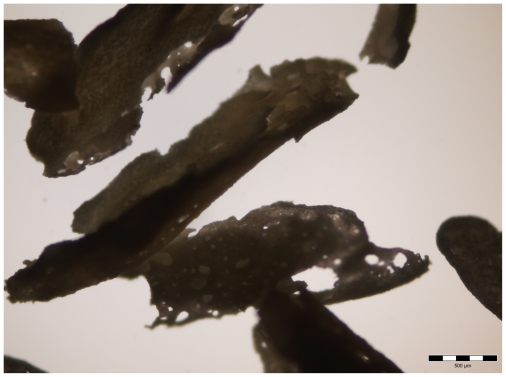
Enlarged macroscopic view of spicules of mineralized tissue recovered by the NaOH hydrolysis of the ectopic bone induction by demineralized incisor implants. Bar represents 500 μm.

Demineralized murine incisor matrix, implanted intramuscularly into syngeneic mice, induces bone formation to varying degrees (Figure 1). The swellings at the sites of MSV inoculation were observed as early as day 3 post inoculation. Tumors grew progressively up to 10 days and then gradually regressed, in most cases until day 16, in a few cases until day 22.

The MSV-induced tumors are pleomorphic and have a high mitotic rate. During the progressive stage of tumor growth, massive destruction of muscle was always observed at the site of virus inoculation, and tumor regression was associated with the regeneration of skeletal muscles, disappearance of neoplastic cells, and the accumulation in the lesion of macrophages, lymphocytes and fibroblasts. Between days 22–28, there was no macroscopic evidence of tumor, and both hind limbs appeared identical.

The yield of mineralized bone induced by implanted demineralized incisors in the sites exposed to MSV tumor during the early phase of induction (virus injection on day 4), was of the same magnitude, as in the non-exposed contralateral site, (0.37 ± 0.34 mg *vs.* 0.46 ± 0.38 mg; difference not significant, t = 1.113).

In contrast, a stimulatory effect was observed when MSV tumor grew during the osteogenic stage of induction, *i.e.*, when MSV was administered on day 12 post incisor grafting, (0.72 mg ± 0.39 *vs.* 0.45 ± 0.34, difference significant at 0.02 < p < 0.05, t = 2.254).

**Figure 2:**
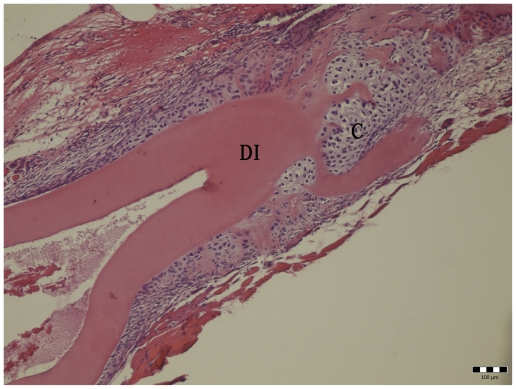
Cartilage (C) formation by demineralized incisor (DI) 10 days post implantation. Hematoxylin-eosin staining. Bar represents 100 μm.

**Figure 3:**
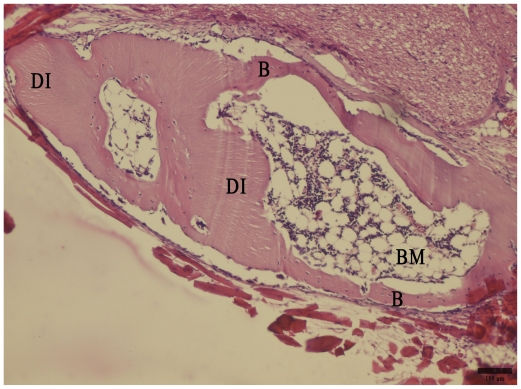
Bone (B) and bone marrow (BM) formed 28 days post demineralized incisor (DI) implantation, not exposed to MSV. Hematoxylin-eosin staining. Bar represents 100 μm.

The yield of bone induction at the contralateral, non-MSV-exposed sites was similar in the 4- and 12-day groups, indicating a lack of systemic effect of MSV-induced tumor (Figures 2 and 3).

**Figure 4:**
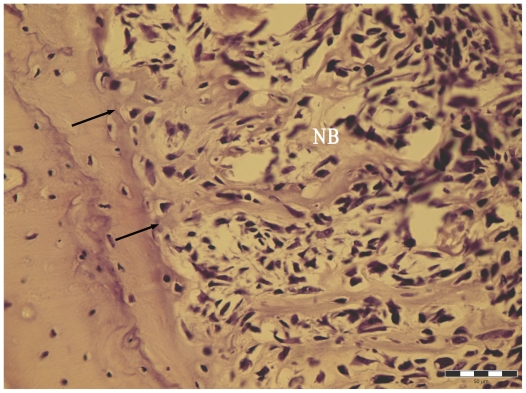
Activation of periosteal membrane of femoral bone at the site of MSV-sarcoma development, 9 days post MSV infection; PAS reaction. Arrows point to the edge of femoral shaft. NB—newly formed bone trabeculae within activated periosteum. Bar represents 50 μm.

In all instances, the exposure of orthotopic femoral bone to MSV sarcoma always activated periosteal osteogenesis (Table 1, Figure 4). The mean increment of dry mass of femoral bones exposed to the MSV-induced sarcoma varied from 3 to 73%, and on average was 17.6% ± 16.7%.

## 4. Discussion

Ectopic cartilage/bone formation by demineralized tooth matrix is a well-known phenomenon [10,11,14], triggered by the release of bone morphogenetic proteins (BMPs) from the implant.

Periosteal bone induction is initiated by cells residing in the periosteal membrane, which are activated by growth factors that are released during immune-mediated spontaneous regression of the tumor MSV-induced [1,28], or by lesions elicited by the T cell mitogen Concanavalin A [29].

The present studies were designed to determine the effect of the development of Moloney sarcoma on bone induction following intramuscular implantation of syngeneic demineralized lower incisors in mice. Murine incisors hydrolyzed for 4–5 hr in chilled 0.6 N HCl were fully demineralized and exhibited good osteoinductive potency. We previously showed that 22 hr demineralization slightly diminishes their osteoinductive power, most likely by affecting their organic phase [26].

We previously histologically examined the early stages of murine demineralized incisor implantation into the thigh muscles, and the earliest cartilage and bone formation was recorded on Day 8. On Day 12, bone trabeculae formed by endochondral or mesenchymal-type osteogenesis were well developed.

Lesions induced by MSV injection during the preosteogenic stage of bone induction (day 4) had no significant effect on ectopic bone yield, although the mean values of bone induction were lower than those for the contralateral, MSV-unexposed implants. In contrast, the exposure of already existing induced bone (12 days) to MSV significantly enhanced the yield of induced bone at those sites.

MSV sarcoma always activated the periosteal membrane of the tumor-adjacent femoral bones to deposit new bone, and the dry mass femoral bone increment varied between 3–78% over the control, and was on average about 18%.

Ectopic bones lack a true periosteum, thus the mechanism of incremental growth of heterotopic bone following MSV-sarcoma exposure must differ from that activating orthotopic bones.

According to Friedenstein [30], osteogenesis in ectopic bones is provided by two different kinds of precursor cell: (1) inductor-dependent clonogeneic cells responsible for maintenance of the mechanocyte cell population—Inducible Osteogenic Precursor Cells, IOPC, and (2) inducer-independent descendants of clonogenic cells—Determined Osteogenic Precursor Cells, DOPC.

We speculate that cytokines or cells generated by the immune reaction which leads to sarcoma rejection are unable to activate recruitment and proliferation of Inducible Osteoprogenitor Cells, the early stage of induction event, but are able to activate Determined Osteoprogenitor Cells descendents of IOPC. Osteoblasts residing in the periosteal membrane of orthotopic bone belong to the DOPC category, thus are susceptible to osteogenic activators.

Another possible explanation for the lack of stimulatory effect on ectopic osteogenesis when MSV sarcoma has developed during an early, preosteogenic stage, is degradation by the tumor or by cells involved in its rejection of BMP released from implanted matrix. Spontaneous regression of MSV sarcoma is a manifestation of a strong cellular response, mainly executed by macrophages, thus the presence of proteolytic enzymes in such lesions is possible. Tumor-associated macrophages release cathepsin, whereas LPS activated peritoneal macrophages release caspases, both are proteolytic enzymes [31,32]. Molecules involved in ectopic bone homeostasis seem to differ in the Con A and in the MSV-evoked inflammatory reactions, as is in the case in chronic inflammatory diseases—rheumatoid-artritis and ankylosing spondylitis [33].

There is a difference in the effect of mediators released by lesions following the T cell mitogen Concanavalin A on ectopic bone by demineralized incisors and by the Moloney sarcoma. The first increases yield of demineralized incisor-induced bone when injected during the preosteogenic stage of induction, an effect not observed when Con A was injected after heterotopic osteogenesis had been established. This might suggests that Con A, in contrast to the Moloney sarcoma, recruits early osteoprogenitor cells, but does not differentiate chondroblasts and osteoblasts. Thus, we postulate that MSV sarcoma does not recruit such cells, but does activate determined cells, as in the case of periosteum activation [1].

## 5. Conclusion

In the model of heterotopic osteogenesis by syngeneic demineralized lower incisors implanted into thigh muscles in mice, the Moloney sarcoma development during the osteogenic stage (day 12 of bone induction), but not during the preosteogenic stage (day 4 of induction) increased the yield of bone induction. This suggests that MSV sarcoma activates determined osteoprogenitor cells, but has minimal, if any, effect on their recruitment.

## Figures and Tables

**Table 1:** The timing effect of MSV-sarcoma development on bone induction following intramuscular implantation of demineralized lower incisor matrix in BALB/c mice, 28 days post implantation.

Time of MSV-inoculation post incisor implantation (day)	Yield of bone induction (mg) at MSV-sarcoma lesions*	Yield of bone induction (mg) at contralateral site, not exposed to MSV*	Yield of femoral bone increment following MSV sarcoma exposure (% of original weight)**
4	(53) 0.37 ± 0.34	(55) 0.46 ± 0.38	(90) 17.6 ± 16.7
12	(32) 0.73 ± 0.39	(32) 0.48 ± 0.34	

* Yield is represented as the mean weight ±S.D. 0.02 < p < 0.05 by Student t-test. In parenthesis is number of implants or paired femoral bones examined.

** % of dry mass gain of the MSV-exposed femora against MSV-unexposed contralateral control. The figures in parenthesis are numbers of mice.
